# Corrigendum to “Effects of continuing nursing intervention on high‐risk patients with diabetic foot ulcers: A meta‐analysis”

**DOI:** 10.1111/iwj.14935

**Published:** 2024-06-03

Xu Z, Wu H, Shi J. Effects of continuing nursing intervention on high‐risk patients with diabetic foot ulcers: A meta‐analysis. Int Wound J. 2024;21(2):e14683. doi:10.1111/iwj.1468310

The affiliations of Hui Wu and Jing Shi are incorrect. It should have been:

**Zhao Xu** ^1^, **Hui Wu** ^2^, **Jing Shi** ^1^

^1^Department of Cardiology and Encephalopathy, Chongqing Liangjiang New Area Hospital of Traditional Chinese Medicine, Chongqing, China

^2^Public Health Division, The Second People's Hospital of Yubei District, Chongqing, China

**Correspondence**: Jing Shi, Department of Cardiology and Encephalopathy, Chongqing Liangjiang New Area Hospital of Traditional Chinese Medicine, No. 3, Nanzhu Road, Dazhulin Street, Liangjiang New District, 401120 Chongqing, China. Email: shijingwuhui_xz@sina.com.

We apologise for this error.

